# Machine learning based association between inflammation indicators (NLR, PLR, NPAR, SII, SIRI, and AISI) and all-cause mortality in arthritis patients with hypertension: NHANES 1999–2018

**DOI:** 10.3389/fpubh.2025.1559603

**Published:** 2025-04-04

**Authors:** Kuijie Zhang, Xiaodong Ma, Xicheng Zhou, Gang Qiu, Chunjuan Zhang

**Affiliations:** Haiyan People’s Hospital, Jiaxing, Zhejiang, China

**Keywords:** inflammation indicators, arthritis, all-cause mortality, prognostic model, neutrophil-percentage-to-albumin ratio, systemic inflammatory response index

## Abstract

**Background:**

This study aimed to evaluate the relationship between CBC-derived inflammatory markers (NLR, PLR, NPAR, SII, SIRI, and AISI) and all-cause mortality (ACM) risk in arthritis (AR) patients with hypertensive (HTN) using data from the NHANES.

**Methods:**

We employed weighted multivariable logistic regression and WQS regression to explore the relationship between inflammatory markers and ACM in AR patients, as well as to determine the weights of different markers. Kaplan–Meier curves, restricted cubic splines (RCS) and ROC curves were utilized to monitor cumulative survival differences, non-linear relationships and diagnostic utility of the markers for ACM risk, respectively. Key markers were selected using XGBoost and LASSO regression machine learning methods, and a nomogram prognostic model was constructed and evaluated through calibration curves and decision curve analysis (DCA).

**Results:**

The study included 4,058 AR patients with HTN, with 1,064 deaths over a median 89-month follow-up. All six inflammatory markers were significantly higher in the deceased group (*p* < 0.001). Weighted multivariable logistic regression showed these markers’ elevated levels significantly correlated with increased ACM risk in hypertensive AR patients across all models (*p* < 0.001). Kaplan–Meier analysis linked higher marker scores to lower survival rates in AR patients with HTN (*p* < 0.001). WQS models found a positive correlation between the markers and hypertensive AR patients (*p* < 0.001), with NPAR having the greatest impact (70.02%) and SIRI next (29.01%). ROC analysis showed SIRI had the highest AUC (0.624) for ACM risk prediction, closely followed by NPAR (AUC = 0.618). XGBoost and LASSO regression identified NPAR and SIRI as the most influential markers, with higher LASSO-based risk scores correlating to increased mortality risk (HR, 2.07; 95% CI, 1.83–2.35; *p* < 0.01). RCS models revealed non-linear correlations between NPAR (Pnon-linear<0.01) and SIRI (Pnon-linear<0.01) with ACM risk, showing a sharp mortality risk increase when NPAR >148.56 and SIRI >1.51. A prognostic model using NPAR and SIRI optimally predicted overall survival.

**Conclusion:**

These results underscore the necessity of monitoring and managing NPAR and SIRI indicators in clinical settings for AR patients with HTN, potentially improving patient survival outcomes.

## 1 Introduction

Arthritis (AR) is a prevalent musculoskeletal disorder characterized by joint pain and functional limitations, primarily including osteoarthritis (OA) and rheumatoid arthritis (RA). By 2040, it is projected that the prevalence of arthritis among adults in the United States will rise to 49% (1). This chronic joint condition often coexists with various comorbidities, particularly cardiovascular (CV) complications, which substantially impair the quality of life and increase mortality rates among affected individuals (HR, 1.49–4.31) (2). For instance, the incidence of cardiovascular disease (CVD) is reported to be 1.5 to 2.0 times higher in RA patients (3), 1.5 times in gout patients (4), and 2 times in those with OA (5). Hypertension (HTN) stands out as a modifiable risk factor for cardiovascular disease, with over half of arthritis patients also suffering from high blood pressure (6). Notably, hypertension has been identified as the strongest risk factor for CVD in individuals with arthritis (7), potentially sharing common pathophysiological pathways such as chronic inflammation, endothelial dysfunction, and arterial stiffness (8–10).

Chronic inflammation is known to compromise and alter vascular function, exacerbating endothelial dysfunction and arterial rigidity, serving as a foundational element in the development and progression of various chronic diseases. It plays a crucial role in the pathogenesis of both AR and HTN (11). Given the substantial burden of hypertension in AR patients and the associated CVD risk, the assessment of inflammation becomes critical. Appropriate inflammatory markers are anticipated to serve as potential biomarkers for evaluating disease severity and all-cause mortality risk in AR patients with HTN.

Recently, various CBC-derived inflammatory markers, including the neutrophil-to-lymphocyte ratio (NLR), platelet-to-lymphocyte ratio (PLR), neutrophil percentage-to-albumin ratio (NPAR), systemic inflammatory response index (SIRI), systemic immune-inflammation index (SII), and aggregate index of systemic inflammation (AISI), have been utilized as prognostic indicators in numerous diseases (12, 13). These biomarkers represent a composite of prognostic parameters, incorporating peripheral blood platelets, lymphocytes, and neutrophils, and they hold significant importance in the diagnosis and management of a range of conditions. Compared to singular inflammatory indicators, CBC-derived markers provide a more comprehensive response to an individual’s immune-inflammatory state and have emerged as promising tools for identifying individuals at high risk of mortality. In our study, we selected these six inflammatory markers based on three key criteria. First, for the pathophysiological relevance, these inflammatory markers reflect systemic inflammation, thrombotic activity and immune status. Second, for the clinical feasibility: These indices are calculated from routine CBC parameters, making them cost-effective for widespread clinical use compared to specialized biomarkers like cytokines. And emerging evidence, for example, elevated NPAR and NLR are independently associated with increased all-cause mortality in community-dwelling heart failure patients (14), while NPAR has shown superior predictive capability for 5-year all-cause mortality in adults with COPD compared to other blood-based inflammatory biomarkers (15). Additionally, increased levels of AISI are significantly related to heightened cardiovascular mortality risk in myocardial infarction patients, serving as an early marker for adverse prognosis (16).

While existing prognostic tools perform well in managing isolated hypertension or arthritis, they significantly deteriorate when applied to HTN-AR comorbidities. For instance, the Framingham Risk Score underestimates cardiovascular mortality by 38% in this population, and RA-specific models fail to account for hypertension-driven endothelial pathology. Meanwhile, conventional cardiovascular risk scores like ASCVD show poor calibration in HTN-AR populations, and no studies have leveraged CBC-derived inflammatory indices for dynamic risk prediction. These gaps highlight the urgent need for dedicated HTN-AR prediction frameworks. Therefore, this study aims to elucidate the association between CBC-derived inflammatory markers and individual all-cause mortality risk in patients with hypertensive AR, analyzing their predictive value for both short-term and long-term mortality and constructing a prognostic model based on data extracted from the National Health and Nutrition Examination Survey (NHANES) spanning 1999 to 2018. This research is intended to provide valuable insights for clinical management.

## 2 Methods

### 2.1 Data source

The National Health and Nutrition Examination Survey (NHANES) database focuses on the comprehensive collection of data related to the health and nutritional status of U.S. households. It includes extensive information such as demographic details, dietary records, physical examination results, questionnaire responses, laboratory data, and restricted data access. The database employs a complex stratified, multi-stage clustered sampling method to ensure that the statistical sample is representative of the entire U.S. population. This study was approved by the Ethics Review Committee of the National Center for Health Statistics, with informed consent obtained from all participants via signed consent forms. Detailed information on the publicly accessible NHANES research design and data can be found at https://www.cdc.gov/nchs/nhanes/.

### 2.2 Study population

This cohort study analyzed data from the continuous NHANES spanning from 1999 to 2018. Individuals under 20 years of age, those with incomplete complete blood count (CBC) parameters, subjects lacking specific information on rheumatoid arthritis (RA) or other forms of arthritis, and individuals with missing essential covariate and follow-up data were excluded from the study. Ultimately, a total of 4,058 patients with arthritis (AR) and comorbid hypertension (HTN) were included. Given that our study incorporated hematological parameters, we utilized Mobile Examination Center (MEC) weights for data analysis. The weight calculation formula for the 1999–2000 and 2001–2002 cohorts was 2/10 × wtmec4yr, while that for the 2003–2018 cohorts was 1/10 × wtmec2yr. Arthritis was defined based on self-reported physician diagnoses from the NHANES database (variable MCQ160A), which does not distinguish between specific subtypes (e.g., rheumatoid arthritis, osteoarthritis), arthritis diagnosis was based on self-reported clinician assessments without subtype classification. Hypertension was determined by any of the following criteria: diagnosed by a physician or healthcare professional; use of antihypertensive medications; or mean systolic blood pressure of at least 140 mmHg and mean diastolic pressure of at least 90 mmHg. Stroke history was recorded as “yes” or “no.”

### 2.3 Definition of inflammatory indices

The inflammatory markers assessed in this study included the neutrophil-to-lymphocyte ratio (NLR), platelet-to-lymphocyte ratio (PLR), neutrophil percentage-to-albumin ratio (NPAR), systemic immune-inflammation index (SII), systemic inflammatory response index (SIRI), and aggregate index of systemic inflammation (AISI), all derived from standard CBC tests. The calculations for these ratios are as follows: NLR = Neutrophil count (NC) / Lymphocyte count (LC);PLR = Platelet count (PC) / Lymphocyte count (LC); SII = Platelet count (PC) × Neutrophil count (NC) / Lymphocyte count (LC); NPAR = Neutrophil percentage (in total WBC count) (%) × 100/Albumin (g/dL); SIRI = Neutrophil count (NC) × Monocyte count (MC) / Lymphocyte count (LC); AISI = Neutrophil count (NC) × Platelet count (PC) × Monocyte count (MC) /Lymphocyte count (LC).

### 2.4 Covariates

The covariates included in this study consisted of age, race, and ethnicity, categorized as follows: Mexican American, other Hispanic, non-Hispanic White, non-Hispanic Black, and other races. Education was classified into three groups: less than high school, high school graduate, and more than high school. Family income was divided into two groups: <1.3 (low income) and > 3.5 (high income). Smoking status was classified as current smokers (defined as having smoked ≥100 cigarettes in their lifetime) versus non-smokers (those who smoked ≤100 cigarettes or never smoked). Alcohol consumption was defined as consuming at least 12 alcoholic drinks in any given year of the participant’s life. Diabetes diagnosis was based on one or more of the following criteria: confirmation by a physician or healthcare professional; fasting blood glucose level of 126 mg/dL or higher; HbA1c percentage of 6.5% or higher; or use of diabetes medications, including insulin.

### 2.5 Statistical analysis

All statistical analyses considered the complex design of NHANES. In the baseline characteristics table, continuous variables were reported as weighted means (95% CI), while categorical variables were reported as weighted percentages (95% CI). Weighted linear regression and weighted chi-square tests were utilized to assess differences between groups.

To explore associations between the six inflammatory markers and the prevalence of all-cause mortality risk in patients with AR and HTN, three logistic regression models were employed: Model 1 included no covariates; Model 2 adjusted for age, gender, race, education level, income, BMI, drinking status, and diabetes status; Model 3 further adjusted for gender in addition to all other covariates. Receiver operating characteristic (ROC) curves were used to evaluate the diagnostic ability of the inflammatory markers (NLR, PLR, NPAR, SII, SIRI, and AISI) for all-cause mortality risk in patients with AR and HTN, and the area under the curve (AUC) values were compared. Additionally, Kaplan–Meier curves were generated to describe the survival probabilities associated with various threshold levels of the inflammatory indices. Trends were estimated by treating the inflammatory markers as continuous variables. We employed weighted quantile sum (WQS) regression models to estimate the joint effect of the six inflammatory indices and identified key indices through WQS scores. Restricted cubic splines (RCS) models assessed the dose–response relationships. Finally, key prognostic indicators were selected via XGBoost and LASSO regression analyses, leading to the construction of a prognostic nomogram model. XGBoost Implementation: nthread = 6, nfold = 5, nrounds = 1,000, verbose = T, early_stopping_rounds = 30. Calibration curves tested the predictive accuracy of the nomogram, and decision curve analysis (DCA) was conducted to evaluate the net benefit of the model for patients.

All analyses were performed using R software version 4.3.3. We utilized the ‘dplyr [1.1.4]’, ‘ggplot2 [3.4.4]’, ‘caret [6.0–94]’, ‘pROC [1.18.0]’, ‘survival [3.3.1]’, ‘survminer [0.4.9]’, ‘rms [6.3.0]’, ‘gWQS [3.0.5]’, xgboost [1.7.8.1] and ‘glmnet [4.1.7]’ packages for data manipulation, visualization, and model building.

## 3 Results

### 3.1 Study cohort selection

**Figure 1:**
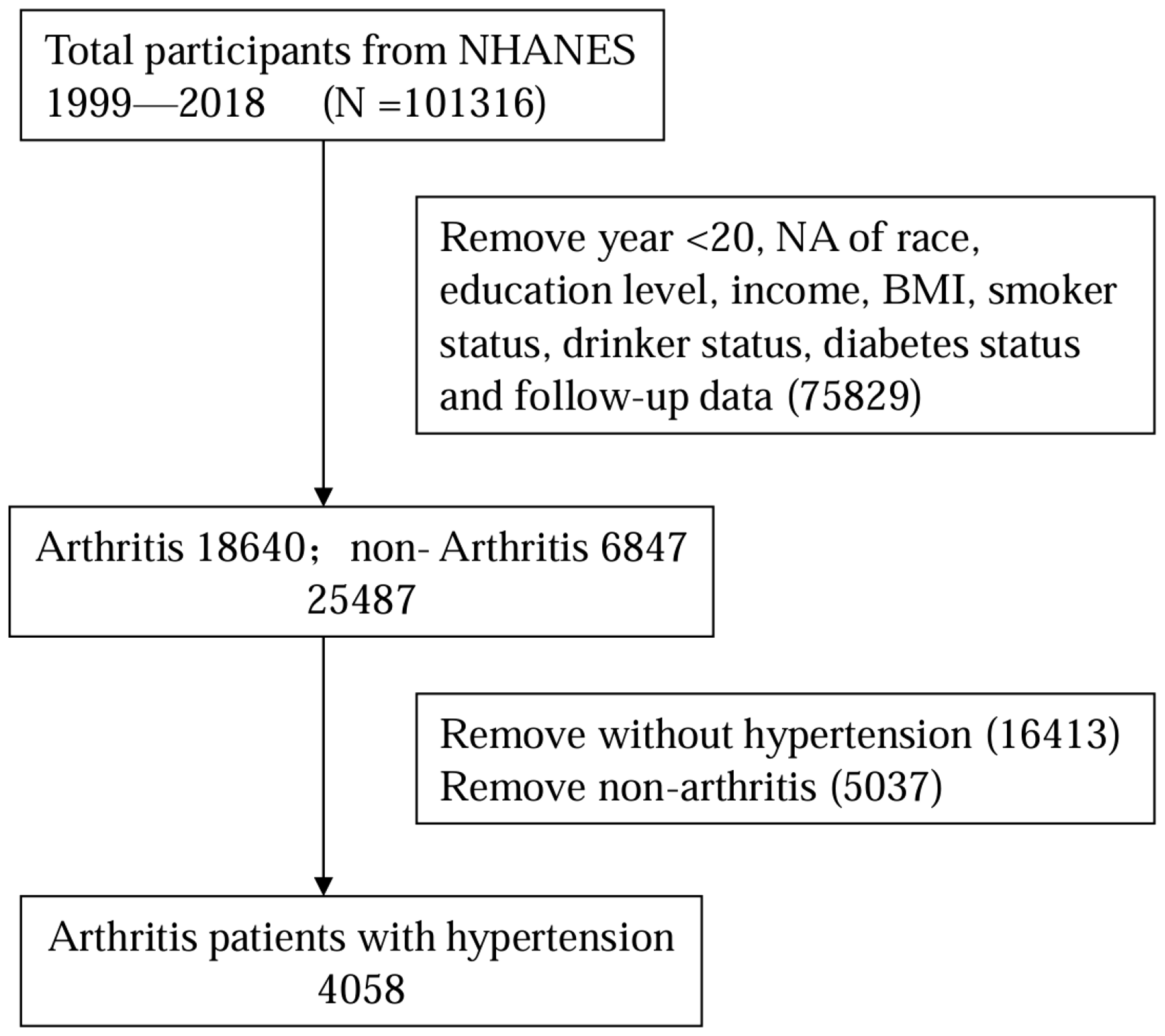
The study flow.

After excluding participants without complete primary variables and mortality status information during follow-up from the NHANES database, a total of 4,058 patients aged 20 years and older with AR combined with HTN were identified. This sample represents a population estimate of 4,606,246 community-dwelling adults in the United States. The flowchart detailing the study inclusion and exclusion process is presented in Figure 1.

### 3.2 Baseline characteristics of the study cohort

Table 1 shows the baseline characteristics of the study cohort. During a median follow-up period of 89 months, there were 1,064 recorded deaths. The deceased participants were significantly older (*p* < 0.001). Aside from smoking status, significant differences were observed between the two groups in terms of sociodemographic, behavioral, and health characteristics. Furthermore, all six inflammatory indices—NLR, PLR, NPAR, SII, SIRI, and AISI—were significantly higher in the mortality group compared to the survival group (*p* < 0.001).

**Table 1:** Baseline characteristics of study participants in arthritis patients with hypertension, weighted.

	Arthritis			
Characteristic	Overall, *N* = 4,058 ^1^	Alive, *N* = 2,994 ^1^	Dead, *N* = 1,064 ^1^	*p* value^2^
Age	63.0 (55.0, 72.0)	61.0 (53.0, 69.0)	73.0 (64.0, 80.0)	**<0.001**
Gender				**0.003**
Female	2,404 (61%)	1863 (63%)	541 (56%)	
Male	1,654 (39%)	1,131 (37%)	523 (44%)	
Race				**<0.001**
Mexican American	412 (3.8%)	350 (4.2%)	62 (2.4%)	
Non-Hispanic Black	1,015 (12%)	786 (12%)	229 (11%)	
Non-Hispanic White	2,129 (77%)	1,433 (75%)	696 (82%)	
Other Hispanic	301 (2.9%)	253 (3.2%)	48 (1.7%)	
Other Race	201 (4.7%)	172 (5.3%)	29 (2.6%)	
Education				**<0.001**
Above high school	1808 (54%)	1,422 (57%)	386 (43%)	
Below high school	539 (7.5%)	359 (6.1%)	180 (12%)	
High school	1711 (39%)	1,213 (37%)	498 (45%)	
Income				**0.041**
Poverty	907 (15%)	676 (14%)	231 (17%)	
Richer	3,151 (85%)	2,318 (86%)	833 (83%)	
BMI	31 (27, 36)	31 (27, 36)	30 (26, 35)	**<0.001**
Drinker	2,663 (71%)	1992 (72%)	671 (64%)	**<0.001**
Smoker	901 (22%)	655 (22%)	246 (23%)	0.3
Diabetes				**<0.001**
Diabetes	1,372 (28%)	956 (26%)	416 (37%)	
Normal	1,242 (36%)	937 (37%)	305 (31%)	
Prediabetes	1,444 (36%)	1,101 (37%)	343 (32%)	
NLR_group				**<0.001**
Q1	1,339 (30%)	1,079 (32%)	260 (22%)	
Q2	1,386 (36%)	1,058 (37%)	328 (32%)	
Q3	1,333 (34%)	857 (31%)	476 (46%)	
PLR_group				**0.003**
Q1	1,339 (31%)	1,024 (32%)	315 (29%)	
Q2	1,382 (35%)	1,050 (37%)	332 (31%)	
Q3	1,337 (34%)	920 (32%)	417 (40%)	
NPAR_group				**<0.001**
Q1	1,339 (33%)	1,093 (36%)	246 (22%)	
Q2	1,380 (36%)	1,046 (36%)	334 (33%)	
Q3	1,339 (32%)	855 (28%)	484 (45%)	
SII_group				**<0.001**
Q1	1,339 (30%)	1,045 (31%)	294 (26%)	
Q2	1,380 (36%)	1,065 (37%)	315 (30%)	
Q3	1,339 (34%)	884 (31%)	455 (45%)	
SIRI_group				**<0.001**
Q1	1,340 (30%)	1,092 (32%)	248 (21%)	
Q2	1,379 (36%)	1,064 (38%)	315 (29%)	
Q3	1,339 (35%)	838 (31%)	501 (49%)	
AISI_group				**<0.001**
Q1	1,340 (30%)	1,062 (31%)	278 (24%)	
Q2	1,379 (35%)	1,071 (37%)	308 (28%)	
Q3	1,339 (35%)	861 (31%)	478 (48%)	

1 Median (Q1, Q3); n (unweighted; %).

2 Design-based Kruskal-Wallis test; Pearson’s X^2: Rao & Scott adjustment. Bold values indicate statistical significance (*P* < 0.05).

### 3.3 Association of six inflammatory indices with all-cause mortality in AR patients with HTN

Results from the weighted multivariable logistic regression analysis in Table 2 indicate that elevated levels of the six inflammatory indices were significantly associated with an increased risk of all-cause mortality in AR Patients with HTN. This association was significant across different models. In Model 1, higher risk was observed in the elevated NPAR group (OR 2.66; 95% CI, 2.10–3.36) and the elevated SIRI group (OR 2.42; 95% CI, 1.87–3.13). In Model 2, which adjusted for age, gender, race, education level, drinking status, and other covariates, the risk remained elevated (OR > 1, *p* < 0.05). In Model 3, after controlling for all covariates including age and gender, the six inflammatory indices continued to show a positive correlation with the risk of all-cause mortality in AR patients with HTN (*p* < 0.001).

**Table 2:** Stepped logistic regression models showing the association between inflammation indicators and the odds of arthritis patients with hypertension.

Indicator	Model 1			Model 2			Model 3		
	OR^1^	95% CI^1^	*p*-value	OR^1^	95% CI^1^	*p*-value	OR^1^	95% CI^1^	*p*-value
NLR									
Q1	—	—		—	—		—	—	
Q2	1.23	0.95, 1.60	0.11	1.12	0.86, 1.48	0.4	1.09	0.83, 1.43	0.5
Q3	2.19	1.73, 2.76	**<0.001**	1.67	1.31, 2.12	**<0.001**	1.60	1.26, 2.02	**<0.001**
PLR									
Q1	—	—		—	—		—	—	
Q2	0.93	0.75, 1.16	0.5	1.05	0.82, 1.34	0.7	1.11	0.87, 1.42	0.4
Q3	1.35	1.09, 1.67	**0.007**	1.41	1.13, 1.75	**0.003**	1.54	1.23, 1.92	**<0.001**
NPAR									
Q1	—	—		—	—		—	—	
Q2	1.53	1.19, 1.97	**0.001**	1.35	1.04, 1.76	**0.023**	1.32	1.01, 1.73	**0.039**
Q3	2.66	2.10, 3.36	**<0.001**	2.27	1.79, 2.88	**<0.001**	2.12	1.68, 2.68	**<0.001**
SII									
Q1	—	—		—	—		—	—	
Q2	0.98	0.78, 1.21	0.8	1.01	0.79, 1.30	>0.9	1.02	0.79, 1.30	0.9
Q3	1.74	1.41, 2.16	**<0.001**	1.87	1.46, 2.39	**<0.001**	1.82	1.42, 2.33	**<0.001**
SIRI									
Q1	—	—		—	—		—	—	
Q2	1.17	0.90, 1.53	0.2	0.97	0.73, 1.29	0.8	0.95	0.71, 1.28	0.7
Q3	2.42	1.87, 3.13	**<0.001**	1.69	1.27, 2.24	**<0.001**	1.56	1.18, 2.07	**0.002**
AISI									
Q1	—	—		—	—		—	—	
Q2	0.96	0.77, 1.20	0.7	0.88	0.69, 1.12	0.3	0.88	0.68, 1.12	0.3
Q3	1.97	1.57, 2.46	**<0.001**	1.80	1.41, 2.29	**<0.001**	1.70	1.33, 2.16	**<0.001**

1 OR, Odds Ratio; CI, Confidence Interval.

Model 1: no covariates were adjusted. Model 2: age, gender, race, education level, income, BMI, drinker status and diabetes status were adjusted. Model 3: age, gender, race, education level, income, BMI, smoker status, drinker status and diabetes status were adjusted.

Bold values indicate statistical significance (*P* < 0.05).

### 3.4 Kaplan–Meier analysis of six CBC-derived inflammatory markers

**Figure 2:**
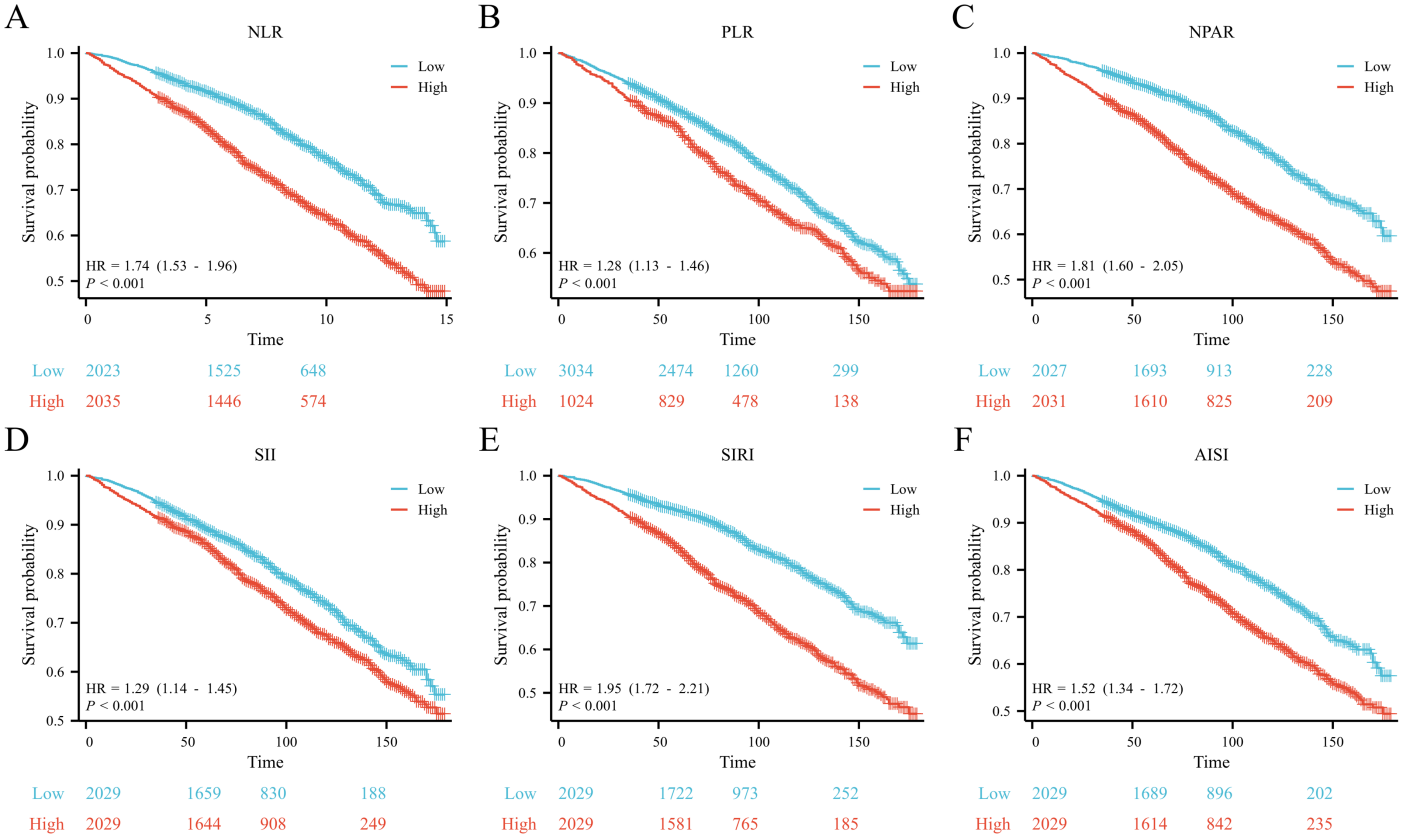
Kaplan–Meier curves for the OS of the **(A)** NLR, **(B)** PLR, **(C)** NPAR, **(D)** SII, **(E)** SIRI and **(F)** AISI.

Kaplan–Meier analysis revealed that patients with higher median levels of the six CBC-derived inflammatory markers had lower survival rates (*p* < 0.001; Figures 2A–F). For instance, the high NPAR group exhibited a hazard ratio (HR) of 1.81 (95% CI, 1.60–2.05, *p* < 0.001; Figure 2C) and the high SIRI group had an HR of 1.95 (95% CI, 1.72–2.21, *p* < 0.001; Figure 2E).

### 3.5 WQS regression model evaluating relationships between six derived inflammatory markers and patients with AR and HTN

**Figure 3:**
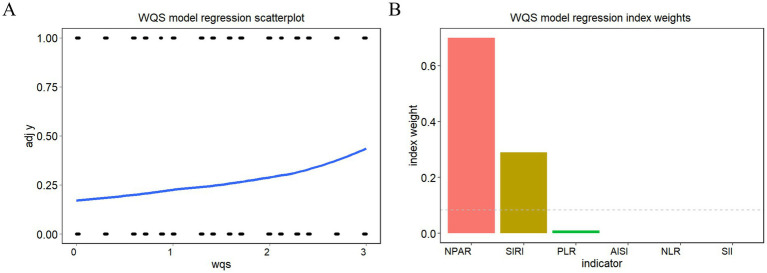
WQS model regression index weights for inflammation indicators on arthritis patients with hypertension **(A)** WQS model regression scatterplot, **(B)** WQS model regression index weights.

The WQS model (Figure 3) demonstrated a positive correlation between the six inflammatory markers and the risk of death in patients with AR and HTN (slope 0.432, *p* < 0.001). The WQS model showed that NPAR had the greatest effect (70.02%), followed by SIRI (29.01%), and PLR (0.97%).

### 3.6 Key CBC-derived inflammatory marker selection

**Figure 4:**
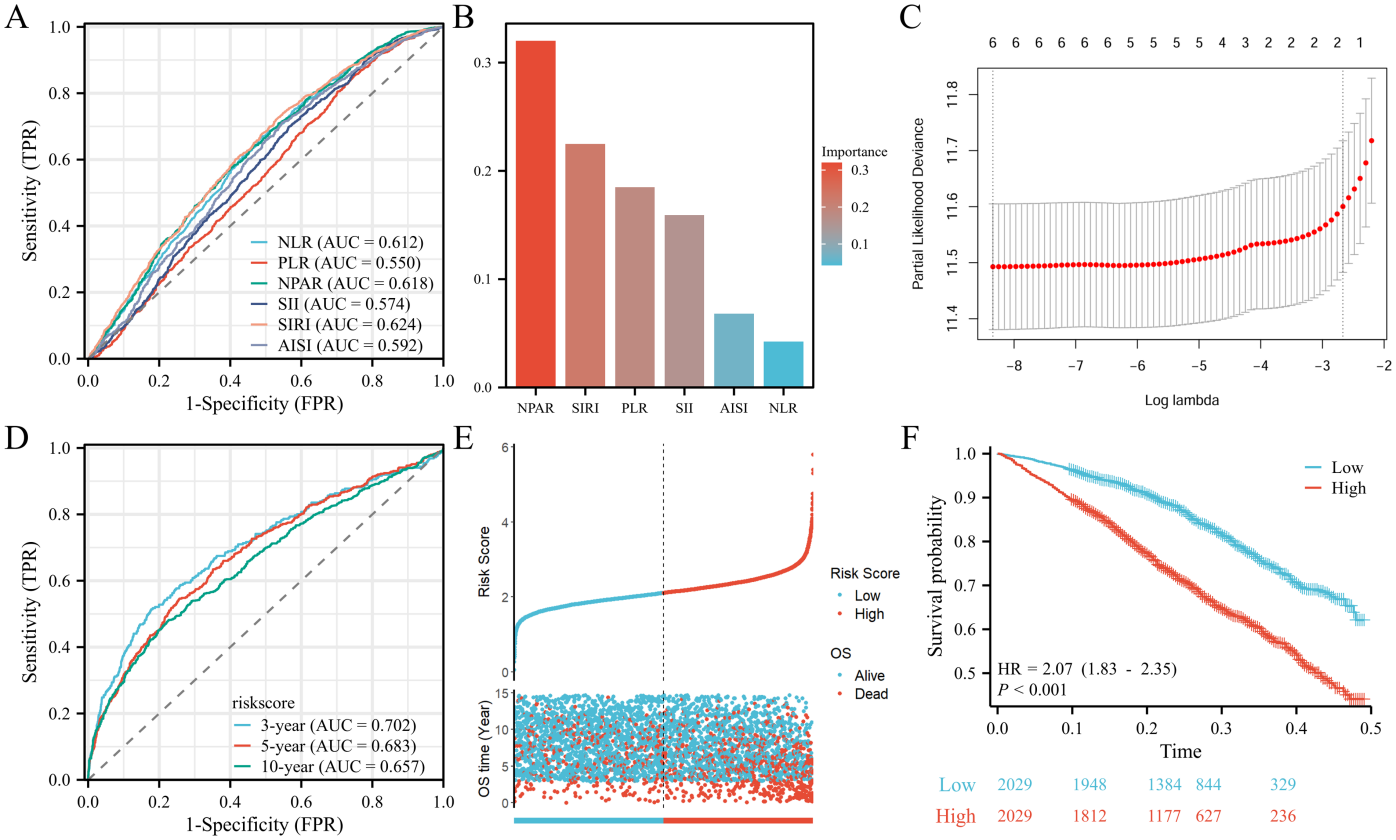
Screening of prognositic biomarkers. **(A)** ROC curves and the AUC values of the six inflammatory markers. **(B)** Screening of diagnostic biomarkers based on XGBoost algorithm. **(C)** LASSO COX regression analysis. **(D)** ROC curves illustrated the predictive efficacy of the risk score for 3-, 5-, and 10-year survival. **(E)** Risk scores distribution and survival status of each patient. **(F)** Kaplan–Meier curves for the OS of the two subtypes.

The predictive performance of the inflammatory indices NLR, PLR, NPAR, SII, SIRI, and AISI for all-cause mortality is illustrated in Figure 4A. Among these indices, SIRI exhibited the highest area under the curve (AUC) value for predicting all-cause mortality risk (AUC = 0.624), closely followed by NPAR (AUC = 0.618). The XGBoost machine learning results for survival risk factor importance (Figure 4B) indicated that NPAR and SIRI had the most significant impact on the outcomes; similar findings were obtained from LASSO regression (Figure 4C). The results of temporal validation and internal random validation consistented with those of the entire dataset, both identified NPAR and SIRI as the top two key indicators (Supplementary Figure 1). Risk scores calculated based on the LASSO regression coefficients led to stratification of patients with AR and HTN into high and low-risk groups based on the median risk score. Time-dependent ROC analyses (Figure 4D) yielded AUC values of 0.702, 0.683, and 0.657 for 3, 5, and 10 years, respectively. The risk factor plots (Figure 4E) demonstrated a positive correlation between higher risk scores and increased mortality risk. Kaplan–Meier analysis revealed that the overall survival of the high-risk group was significantly shorter than that of the low-risk group, indicating a poorer prognosis for the high-risk cohort (HR, 2.07; 95% CI, 1.83–2.35; *p* < 0.01; Figure 4F).

### 3.7 Nonlinear relationship between NPAR/SIRI and all-cause mortality in AR patients with HTN

**Figure 5:**
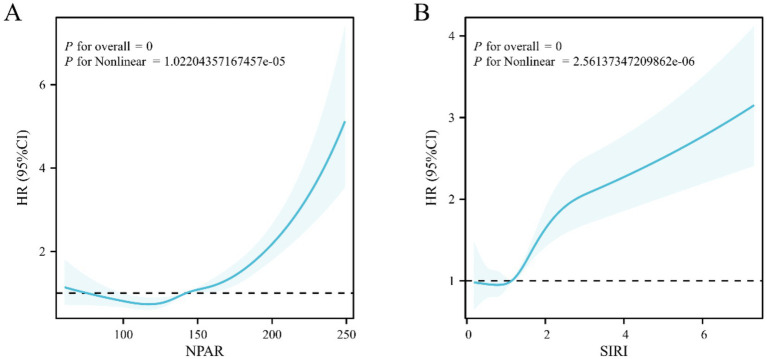
RCS analysis depicting the relationship between inflammation indicators and all-cause mortality in arthritis patients with hypertension **(A)** NPAR, **(B)** SIRI.

The results of the restricted cubic splines (RCS) model (Figure 5) indicated a nonlinear correlation between NPAR (Pnonlinear <0.01) and SIRI (Pnonlinear <0.01) and the risk of all-cause mortality in AR patients with HTN. Specifically, mortality risk sharply increased when NPAR exceeded 148.56 or SIRI exceeded 1.51.

### 3.8 Development of prognostic models for all-cause mortality risk in patients with AR and HTN

**Figure 6:**
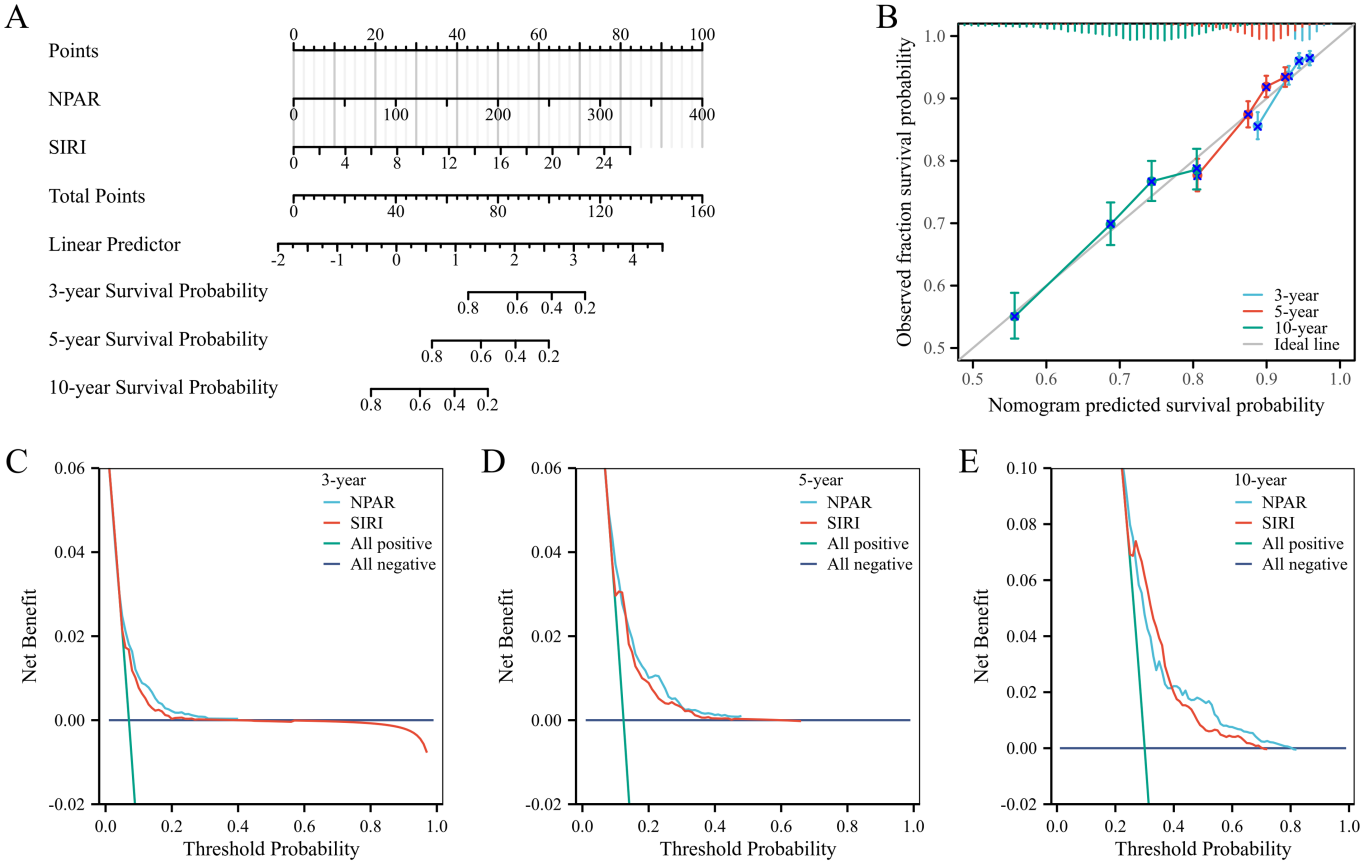
**(A)** A nomogram of risk model containing NPAR and SIRI; **(B)** The calibration plot of nomogram. **(C)** The DCA curves of nomogram for 3 years. **(D)** The DCA curves of nomogram for 5 years. **(E)** The DCA curves of nomogram for 10 years.

Based on key findings from the index selection process, a prognostic nomogram was developed utilizing NPAR and SIRI to predict overall survival (OS) at 3, 5, and 10 years (Figure 6A). The C-index for the prognostic model was 0.637 (95% CI, 0.627–0.647). Calibration curve analysis showed good consistency between observed and predicted rates for 3-, 5-, and 10-year overall survival (Figure 6B). Decision curve analysis (DCA) demonstrated that the comprehensive model incorporating the two inflammatory indices provided optimal net benefits for predicting 3-, 5-, and 10-year overall survival compared to other options (i.e., predictions based on each factor alone; Figures 6C–E).

## 4 Discussion

Patients with arthritis (AR) complicated by hypertension (HTN) are at a higher risk of all-cause mortality and often experience poor prognosis, making the search for appropriate prognostic markers crucial. This study investigated the relationship between six complete blood count (CBC)-derived inflammatory markers and the risk of all-cause mortality in AR patients with HTN. Unlike previous studies that primarily examined individual inflammatory markers, our analysis, based on the NHANES database, analyzed the cumulative effect of multiple inflammatory indicators, providing a more comprehensive understanding of their impact on patient outcomes. We revealed a significant positive correlation between high levels of six inflammatory markers and increased risk of all-cause mortality in hypertensive arthritis patients, indicating that inflammation plays a key role in the prognosis of this condition, independent of potential demographic, socioeconomic, and lifestyle confounders. Notably, the WQS model identified NPAR as the most significant contributor to mortality risk, followed by SIRI. This result was validated in XGB and LASSO machine learning, where NPAR and SIRI emerged as independent predictors of survival time in AR patients with HTN (*p* < 0.01). Using LASSO regression coefficients to calculate risk scores and dividing AR patients with HTN into high and low-risk subgroups, we found that higher risk scores were positively correlated with increased mortality risk (HR, 2.07, 1.83–2.35, *p* < 0.01). Finally, a prognostic nomogram based on NPAR and SIRI was constructed, showing the best net benefit for predicting total survival time over 3, 5, and 10 years. These results underscore the necessity of monitoring and managing NPAR and SIRI indicators in clinical settings for AR patients with HTN, potentially improving patient survival outcomes.

The logistic regression analysis results of the six CBC-derived inflammatory markers showed a significant positive correlation between elevated inflammatory markers and the risk of all-cause mortality in AR patients with HTN, indicating a key role of inflammation in the prognosis of these patients, consistent with previous findings. For instance, Zhou et al. found that (17) higher NLR could independently predict long-term mortality risk in US adults with RA and could serve as an inexpensive and widely available prognostic marker in RA; Jiang found that (18) SIRI could be used as a new inflammatory marker for disease activity in gouty arthritis; Liu found (19) a positive correlation between SII and rheumatoid arthritis (OR = 1.167, 95% CI = 1.025–1.328, *p* = 0.020); Yin found that (20) elevated SII levels independently predicted the risk of death from all causes and cardiovascular-specific mortality in RA patients; Wang revealed (21) a nonlinear positive correlation between the inflammatory biomarker SII and all-cause mortality and cardiovascular mortality in RA patients, suggesting that clinicians should be vigilant about adverse outcomes when SII levels exceed the threshold of 529.7, providing a new method for clinicians to quickly identify RA patients at higher risk of death. Yin found (22) a statistically significant association between Ln(AISI) levels and increased odds of RA (odds ratio [OR]: 1.097; 95% confidence interval [CI]: 1.096–1.099, *p* < 0.001), suggesting the potential of AISI as an innovative, important, and suitable inflammatory biomarker for predicting the risk of RA in older adult Americans; Wang (23) found a nonlinear positive correlation between SIRI and all-cause mortality and cardiovascular mortality in RA patients; Zhou (24) found that patients with elevated SII and SIRI had an 88 and 67% increased risk of cardiovascular death, respectively. These studies focused on the correlation between single inflammatory markers and the risk of onset or death in AR, without comprehensive analysis of multiple inflammatory indicators, and the correlation between NPAR and the risk of all-cause mortality in AR patients with HTN has not been previously reported.

In our study, several statistical models, including weighted multivariate logistic regression and WQS regression analysis, were used to investigate the relationship between inflammatory markers and the risk of all-cause mortality in patients with hypertension and arthritis. The results from weighted multivariate logistic regression analysis and KM analysis across different models revealed a significant positive correlation between elevated levels of six inflammatory markers and increased risk of all-cause mortality in this patient population. Additionally, ROC analysis showed that SIRI had the highest AUC value for predicting the risk of all-cause mortality (AUC = 0.624), followed by NPAR (AUC = 0.618); the application of the WQS model also found that NPAR was the most significant contributor to mortality risk, accounting for nearly 70.02% of the effect, followed by SIRI, with an effect proportion of 29.01%. These findings were further supported in XGB and LASSO machine learning, which demonstrated a positive linear relationship between specific inflammatory exposures and mortality outcomes from different analytical perspectives, enhancing the robustness of our conclusions and emphasizing the importance of inflammatory markers as potential intervention targets in managing the mortality risk of AR patients with HTN.

Inflammation leads to increased counts of neutrophils, monocytes, and platelets, and decreased lymphocyte counts, making their ratios important tools for indirectly assessing inflammatory status and cell-mediated immunity. AR patients with HTN are in a chronic inflammatory state, leading to abnormal activation of the immune system and excessive release of cytokines, making the body’s inflammatory status more complex and the long-term risk of death higher. Therefore, inflammatory markers of single cell subtypes may not be sufficient to reflect the complexity and severity of the immune-inflammatory status of these patients. SIRI, a novel inflammatory marker that integrates counts of neutrophils, lymphocytes, and monocytes, can reflect the status of three inflammatory cells at the same time and more comprehensively reflect the body’s inflammatory status and immune balance than using MLR, NLR, or PLR alone, and is considered a better new inflammatory index than MLR and NLR (25, 26). A high SIRI state can reflect a strong pro-inflammatory response mediated by monocytes and neutrophils and a weak or suppressed anti-inflammatory response mediated by lymphocytes, and monocytes play an important role in the mechanism of AR, which may be one of the reasons why SIRI has more advantages in predicting the risk of all-cause mortality in AR patients with HTN (18). NPAR integrates the proportion of neutrophils and the concentration of albumin, two different biomarkers that reflect acute and chronic inflammation (27), respectively, providing a more comprehensive assessment. This ratio has shown correlations with various cardiovascular diseases (28, 29) and metabolic disorders (30, 31). As an emerging biomarker, NPAR can more accurately represent the inflammatory state and may more effectively reflect the inflammatory state (14) than traditional markers such as albumin, neutrophil percentage, and NLR. Unlike lymphocyte counts, albumin is less affected by acute fluctuations, which may make NPAR and SIRI more stable chronic inflammation indicators (32). For example, Chou’s (15) study on inflammatory markers of mortality in chronic obstructive pulmonary disease confirmed that both NPAR and NLR could predict mortality, but NPAR had better predictive performance. Our findings expand previous studies on inflammatory markers and AR, finding that six CBS-derived markers are all related to the risk of all-cause mortality in AR patients with HTN, but SIRI and NPAR have potential advantages.

The integration of NPAR and SIRI into routine clinical practice presents a pragmatic strategy for risk stratification and personalized management in hypertensive arthritis patients. Our findings support a two-pronged approach: First, dynamic risk stratification using validated thresholds (NPAR>148.56/SIRI>1.51) identifies high-risk patients warranting intensified surveillance, including 3-month multidisciplinary evaluations with echocardiography and cytokine profiling, consistent with EULAR’s 2023 comorbidity monitoring guidelines. Second, mechanism-driven therapeutics prioritize IL-6 inhibitors (e.g., tocilizumab) over conventional DMARDs in high-risk subgroups, capitalizing on their dual endothelial protection and anti-inflammatory effects (33), while substituting ACE inhibitors with ARNI (sacubitril/valsartan) aligns with ACC/AHA’s inflammatory hypertension management algorithms. Crucially, these indices exploit ubiquitously available complete blood count (CBC) parameters, enabling EHR-embedded real-time risk recalibration without additional healthcare expenditures - a critical advantage in resource-constrained settings. This paradigm shift from reactive to preemptive care exemplifies the “precision public health” mandate, potentially reducing cardiovascular mortality in HTN-AR populations through timely intervention.

Our XGBoost-based model addresses these challenges through three innovations: (a) explicit modeling of neutrophil-endothelial interactions via NPAR/SIRI; (b) machine learning-powered nonlinear risk calibration; and (c) EHR-integratable thresholds enabling real-world implementation. This represents a paradigm shift from reactive to preemptive care, moving beyond the limitations of current tools to provide more accurate and actionable prognostic insights for HTN-AR patients. However, some limitations must be acknowledged. The six CBC-derived biomarkers were not included as time-dependent variables, as they may dynamically change during follow-up, and using only one measurement may introduce bias. Second, we were unable to differentiate between subtypes of arthritis, which warrants further validation in clinically confirmed cohorts. Nevertheless, this study has several important strengths. First, the current analysis used data from NHANES, which comes from a large and diverse sample of the US population. Study participants represent AR patients with HTN in the community, and these findings are considered generalizable to the entire US population. Second, the study employed multiple analytical methods to assess the prognostic role of inflammatory biomarkers and carefully adjusted for multiple demographic and lifestyle variables that are rarely considered in hospital settings and constructed a prognostic model, with robust results providing a reference for clinical risk management of AR patients with HTN.

## 5 Conclusion

In conclusion, this study demonstrates that CBC-derived inflammatory markers, particularly NPAR and SIRI, are significantly associated with increased mortality risk in AR patients with HTN. The prognostic model developed using these two markers has predictive value for all-cause mortality risk and can serve as clinical management biomarkers for this patient population, providing information for clinical decision-making and facilitating more targeted and effective management strategies.

## Data Availability

The datasets presented in this study can be found in online repositories. The names of the repository/repositories and accession number(s) can be found at: https://wwwn.cdc.gov/nchs/nhanes/default.aspx.

## Supplementary material

Supplementary Figure 1 The XGBoost results of temporal validation with **(A)** 1999-2008 sets and **(B)** 2009-2018 sets; internal random validation with **(C)** training sets and **(D)** validation sets.
