# Supplementary figures and images for: Machine learning based association between inflammation indicators (NLR, PLR, NPAR, SII, SIRI, and AISI) and all-cause mortality in arthritis patients with hypertension: NHANES 1999–2018
Source: Front Public Health. 2025 Apr 4;13:1559603. doi: 10.3389/fpubh.2025.1559603 (PMC12007114; doi:10.3389/fpubh.2025.1559603)

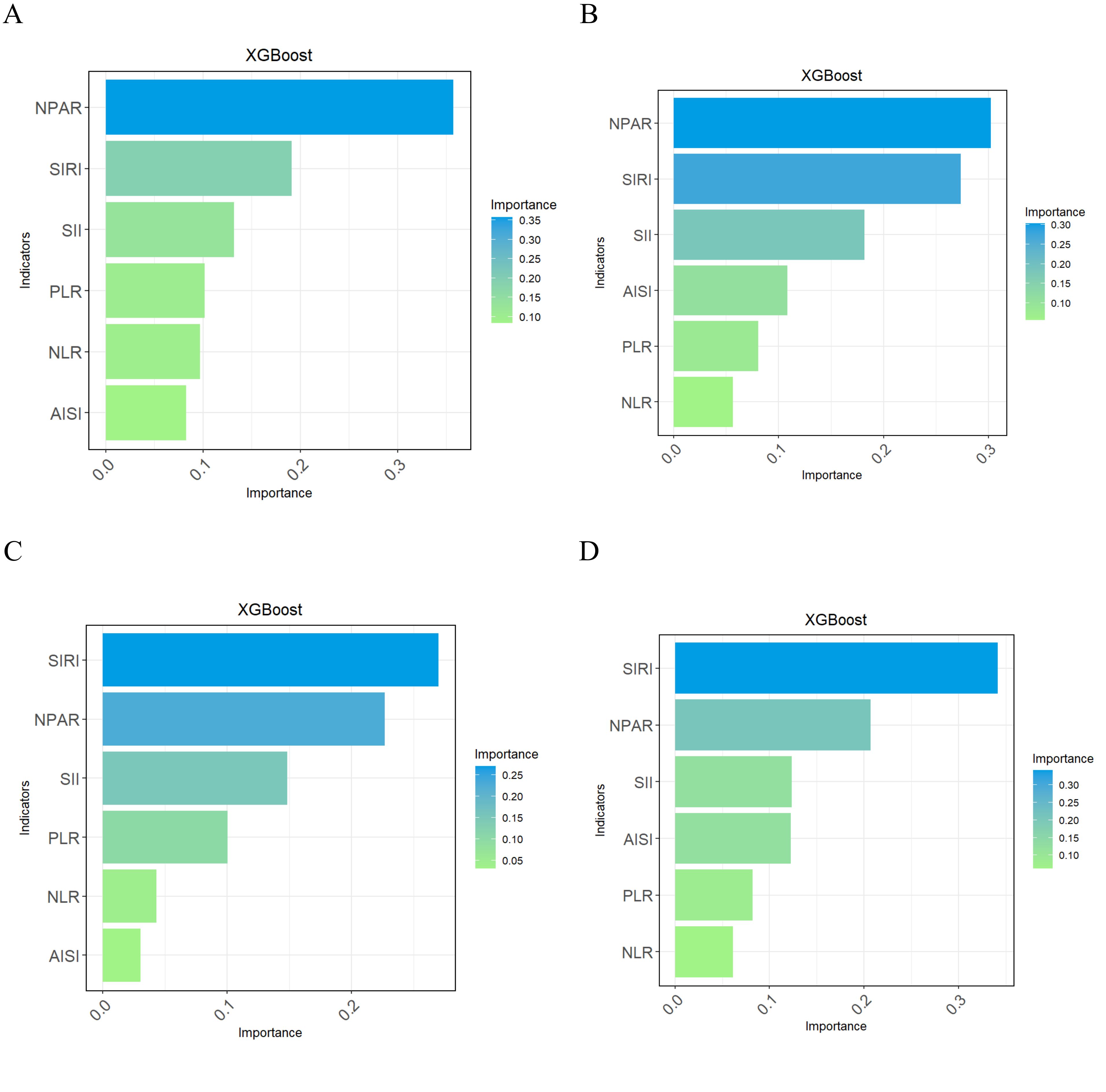

Supplement: Supplementary Figure 1 — The XGBoost results of temporal validation with (A) 1999-2008 sets and (B) 2009-2018 sets; internal random validation with (C) training sets and (D) validation sets. [file Image_1.tiff]
